# Supplementary material for: Generation of precision microstructures based on reconfigurable photoresponsive hydrogels for high-resolution polymer replication and microoptics
Source: Nat Commun. 2024 Jul 6;15:5673. doi: 10.1038/s41467-024-50008-6 (PMC11227548; doi:10.1038/s41467-024-50008-6)
Supplement: Supplementary file 1 — Supplementary Information [file 41467_2024_50008_MOESM1_ESM.pdf]

# Supplementary Information

# Generation of Precision Microstructures Based on Reconfigurable Photoresponsive Hydrogels for High-Resolution Polymer Replication and Microoptics

Pang Zhu[1], Qingchuan Song[1,2], Sagar Bhagwat[1], Fadoua Mayoussi[1], Andreas Goralczyk[1], Niloofar Nekoonam[1], Mario Sanjaya[3], Peilong Hou[1], Silvio Tisato[4], Frederik Kotz-Helmer[1,2,3], Dorothea Helmer[1,2,3,4*], Bastian E. Rapp[1,2,3,4]

1. Laboratory of Process Engineering, NeptunLab, Department of Microsystems Engineering (IMTEK), Albert Ludwig University of Freiburg, 79110 Freiburg, Germany.
2. Freiburg Center of Interactive Materials and Bioinspired Technologies（FIT）, Albert Ludwig University of Freiburg, 79110 Freiburg, Germany.
3. Glassomer GmbH, 79110 Freiburg, Germany.
4. Freiburg Materials Research Center (FMF), Albert Ludwig University of Freiburg, 79104 Freiburg, Germany.

* Corresponding author: dorothea.helmer@neptunlab.org

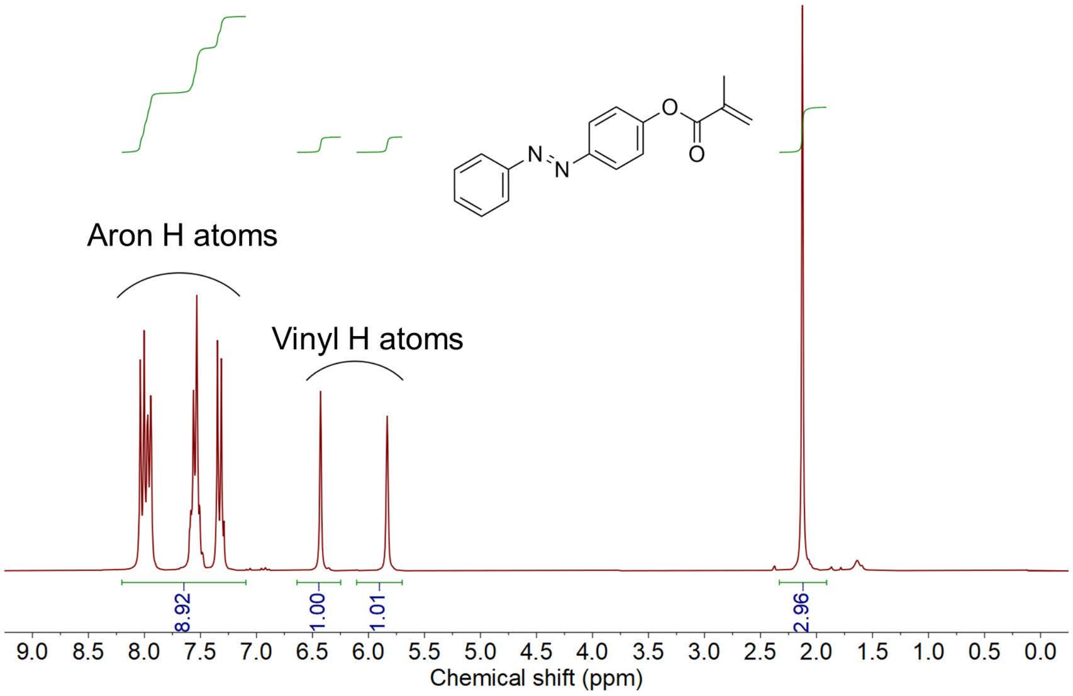


**Supplementary Fig. 1** $^{1}H$ NMR spectra of 4-methacryloyloxy azobenzene monomer. $CDCl_3$ was used as the solvent.

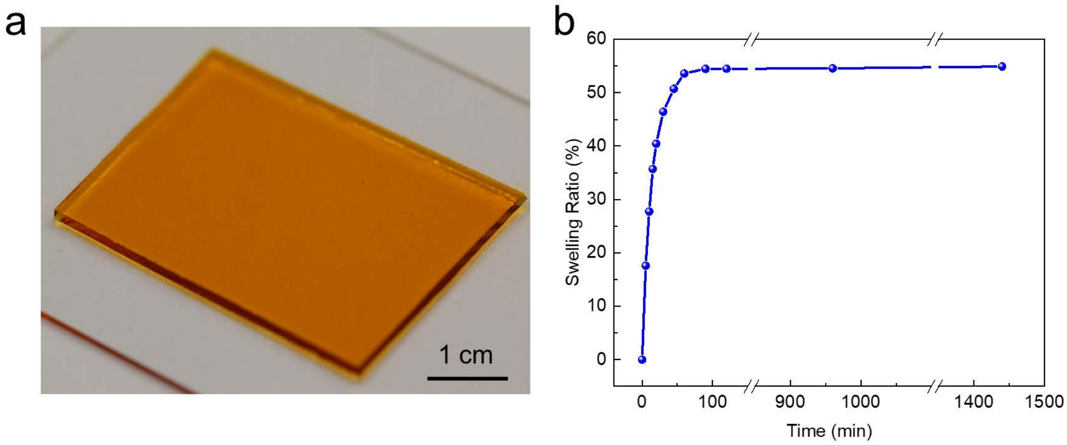


**Supplementary Fig. 2** Optical picture (a) and swelling curve (b) of AM/AZO-CD gel as a function of time.

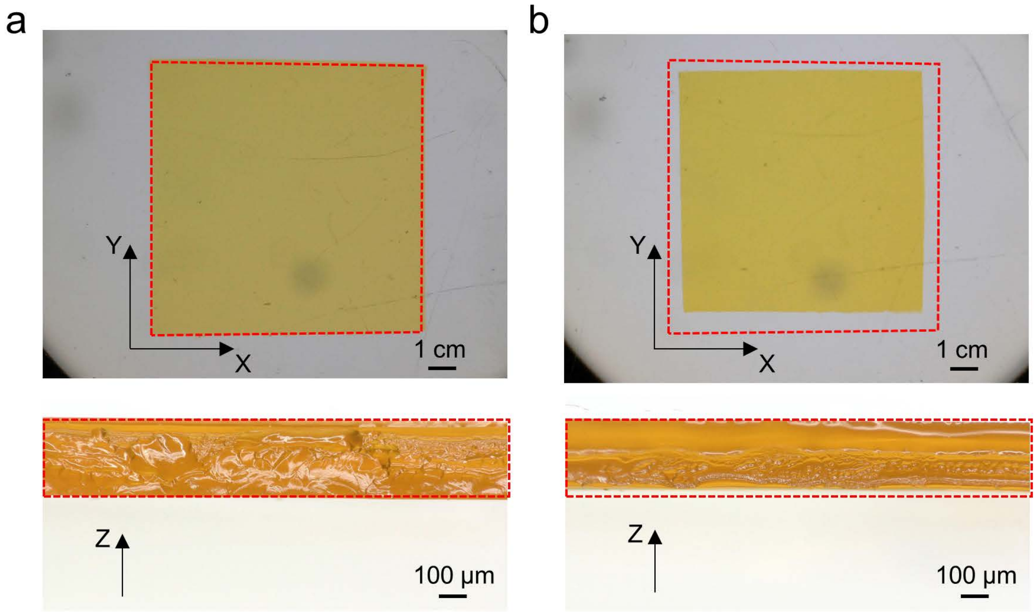


**Supplementary Fig. 3** Optical pictures of reversible AM/AZO-CD hydrogel under (a) expanded state and (b) shrunken state.

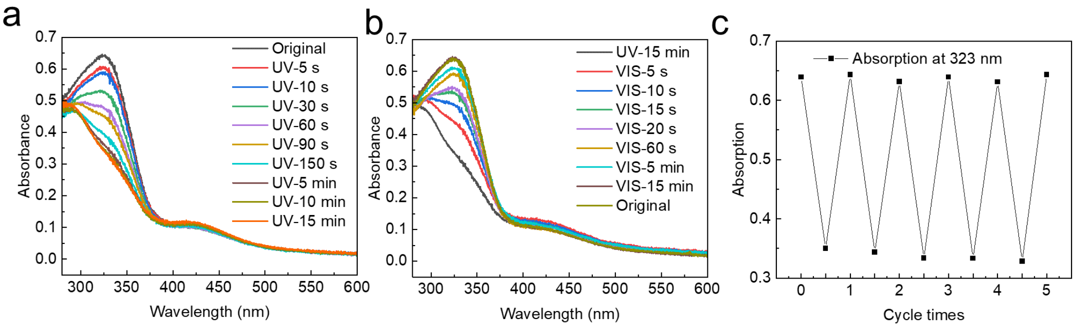


**Supplementary Fig. 4** (a) Absorption spectrum of the original AM/AZO-CD hydrogel at different UV irradiation times; (b) Absorption spectrum of AM/AZO-CD hydrogel at different VIS irradiation time after being firstly exposed to UV for 15 min; (c) The absorption peak at 323 nm during five UV-VIS irradiation cycles showing almost no degradation over 5 full cycles.

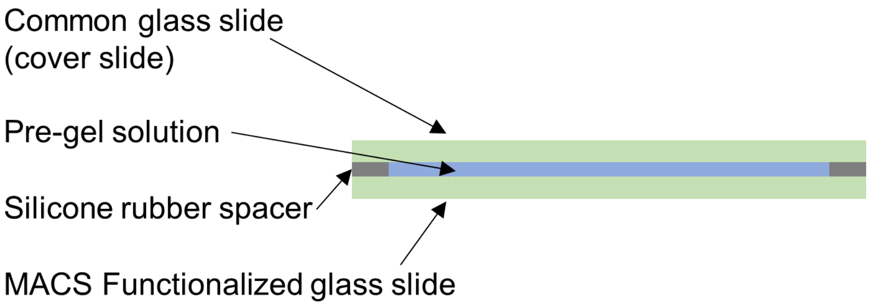


**Supplementary Fig. 5** Setup for the AM/AZO-CD gel polymerization.

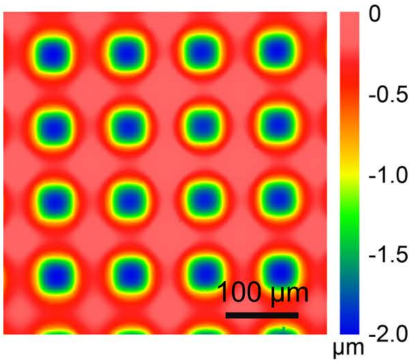


**Supplementary Fig. 6** Profile of square arrays on the micromold display surface characterized 1 h after UV irradiation.

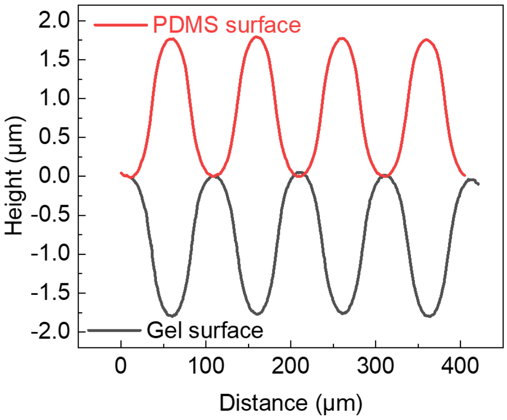


**Supplementary Fig. 7** Cross sectional profiles of the hydrogel (black) and replicated PMDS (red) shows that the structure in the hydrogel surface was replicated accurately.

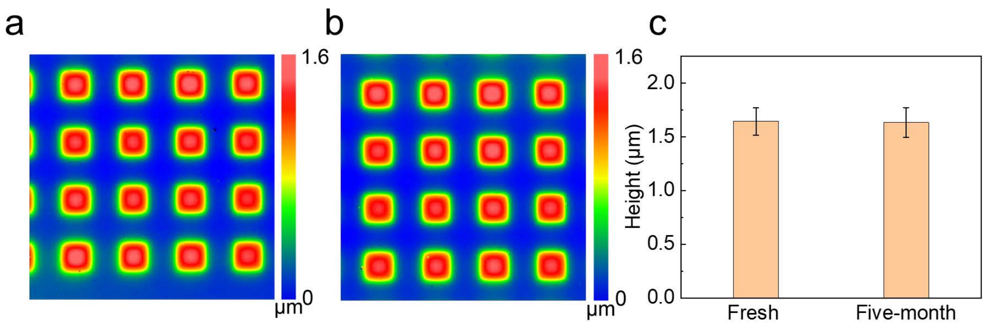


**Supplementary Fig. 8** WLI images of PDMS microstructures replicated from a fresh (a) and a five-month stored (b) hydrogel, and (c) corresponding statistical analysis of height distribution. Data in c are presented as mean values ± SD. Error bars represent the standard deviation from three samples.

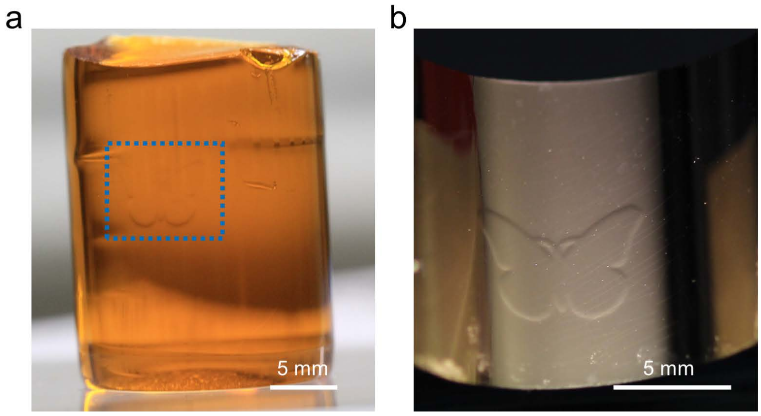


**Supplementary Fig. 9** (a) The picture of the cylindrical hydrogel with an engraved butterfly structure and (b) the picture of the PDMS with replicated butterfly on the curved surface.

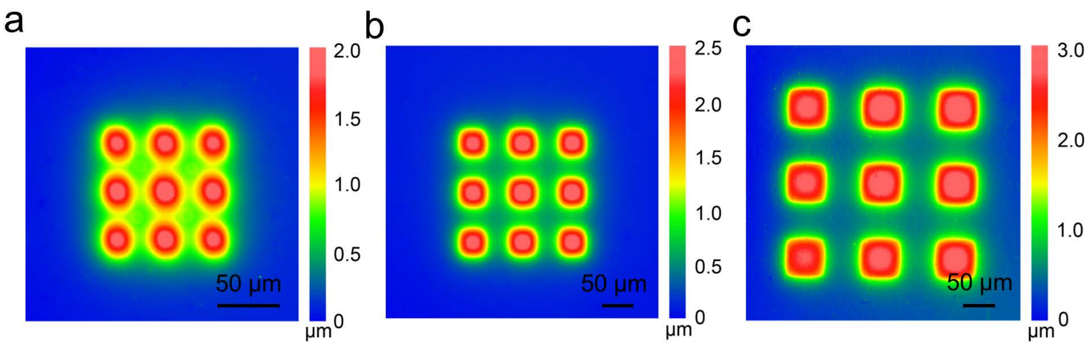


**Supplementary Fig. 10** Lateral resolution test of the micromold display using maskless projection lithography. Square array patterns with various width/gap distances: (a) 20 μm/20 μm, (b) 40 μm/40 μm, (c) 60 μm/60 μm.

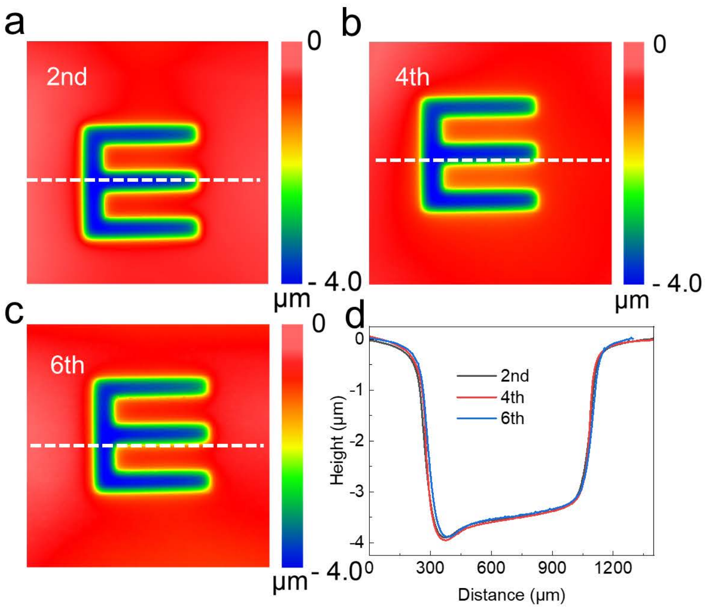


**Supplementary Fig. 11** Multiple structuring-erasing cycles on the hydrogel without PDMS replication. (a-c) WLI images of structured hydrogel surface and (d) corresponding profiles of corresponding microstructures.

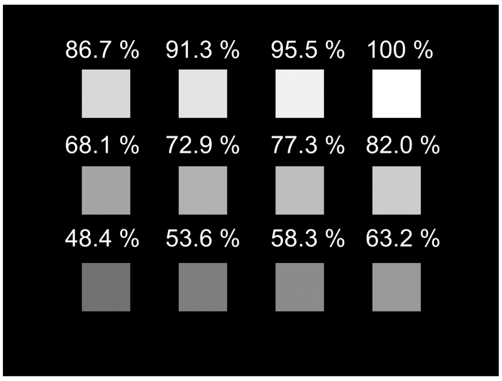


**Supplementary Fig. 12** Transparency analysis of grayscale mask employed in DMD grayscale lithography.

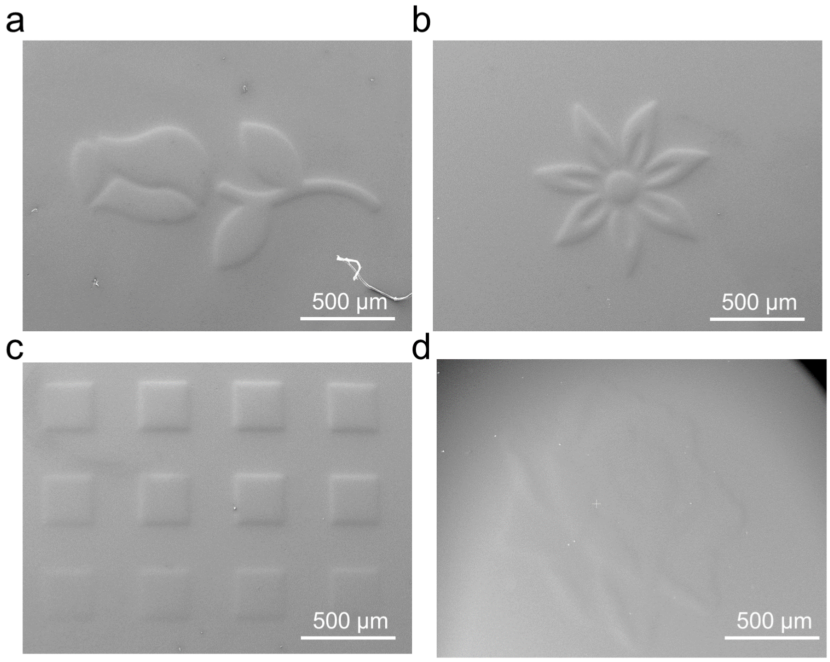


**Supporting Fig. 13** SEM images of samples prepared via maskless grayscale projection lithography shown in Fig. 3e, 3f, 3h, and 3k.

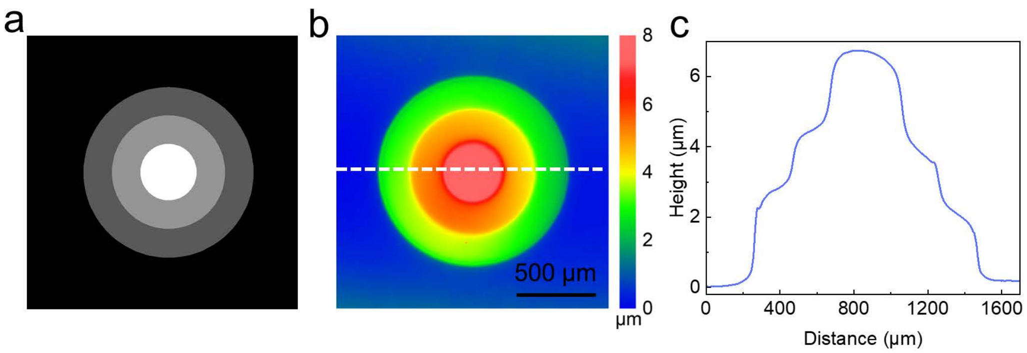


**Supplementary Fig. 14** (a) An annular digital mask used for grayscale lithography, and (b) WLI image and (c) feature profile of obtained staircase structure with three height levels on the replicated PDMS.

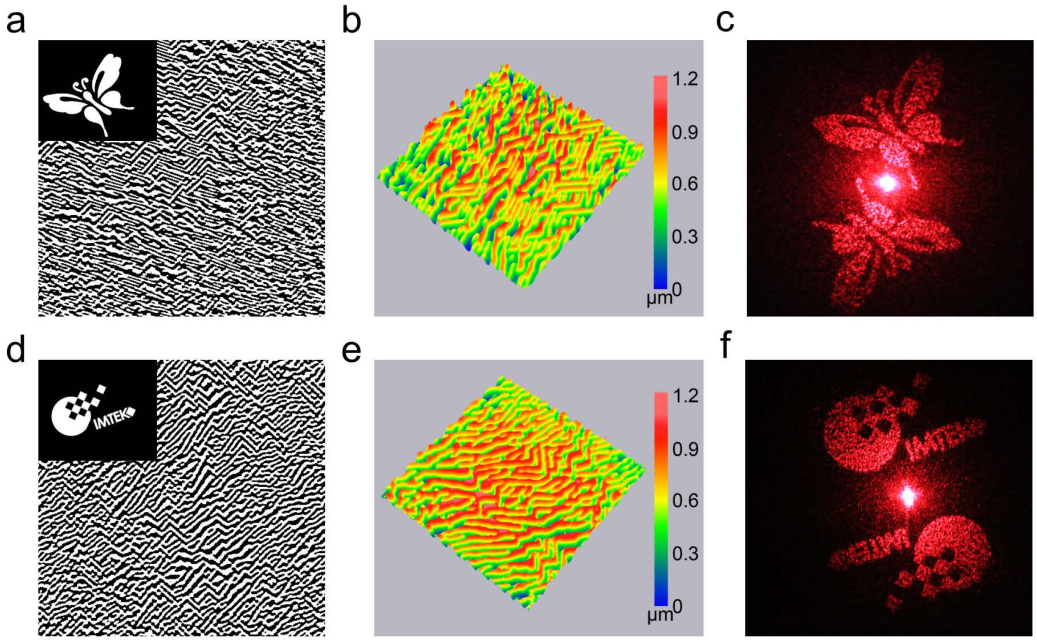


**Supplementary Fig. 15** (a & d) Binary holograms used as digital masks for lithography using the Gerchberg-Saxton (GS) algorithm (insets are original user-designed images to generate corresponding digital masks); (b & e) 3D profiles of the replicated PDMS DOE devices determined by WLI which show a feature resolution of about 30 µm and (c & f) corresponding diffraction patterns;

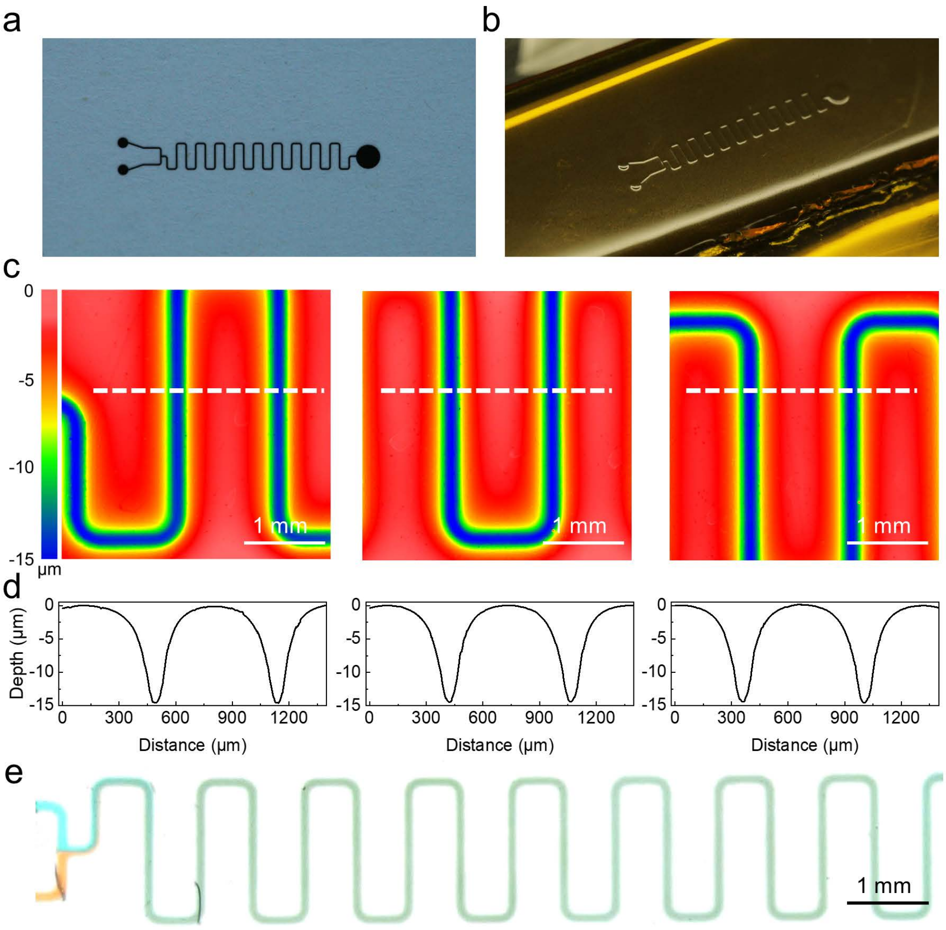


**Supplementary Fig.16**. Fabrication of microchannel using a negative mask by one-step replication. (a) The negative mask employed for engraving convex microstructure on the hydrogel surface; (b) The optical image of hydrogel with convex microstructure; (c) WLI characterization and (d) feature profile of replicated microchannel in PDMS at different locations along the channel; (e) Optical picture of the microchannel filled with dyed water showing diffusive mixing.
